# Reversible Disassembly of the Actin Cytoskeleton Improves the Survival Rate and Developmental Competence of Cryopreserved Mouse Oocytes

**DOI:** 10.1371/journal.pone.0002787

**Published:** 2008-07-30

**Authors:** Basarab G. Hosu, Steven F. Mullen, John K. Critser, Gabor Forgacs

**Affiliations:** 1 Department of Molecular and Cellular Biology, Harvard University, Cambridge, Massachusetts, United States of America; 2 21st Century Medicine, Fontana, California, United States of America; 3 Department of Biological Sciences, University of Missouri, Columbia, Missouri, United States of America; 4 Department of Veterinary Pathobiology, University of Missouri, Columbia, Missouri, United States of America; 5 Department of Physics, University of Missouri, Columbia, Missouri, United States of America; University of California, Berkeley, United States of America

## Abstract

Effective cryopreservation of oocytes is critically needed in many areas of human reproductive medicine and basic science, such as stem cell research. Currently, oocyte cryopreservation has a low success rate. The goal of this study was to understand the mechanisms associated with oocyte cryopreservation through biophysical means using a mouse model. Specifically, we experimentally investigated the biomechanical properties of the ooplasm prior and after cryopreservation as well as the consequences of reversible dismantling of the F-actin network in mouse oocytes prior to freezing. The study was complemented with the evaluation of post-thaw developmental competence of oocytes after *in vitro* fertilization. Our results show that the freezing-thawing process markedly alters the physiological viscoelastic properties of the actin cytoskeleton. The reversible depolymerization of the F-actin network prior to freezing preserves normal ooplasm viscoelastic properties, results in high post-thaw survival and significantly improves developmental competence. These findings provide new information on the biophysical characteristics of mammalian oocytes, identify a pathophysiological mechanism underlying cryodamage and suggest a novel cryopreservation method.

## Introduction

Assisted reproductive technologies have become not only a solution for millions of infertile couples worldwide, but also an essential tool for transgenic animal production [1], embryonic stem cell research and reproductive or therapeutic cloning [2]. The number of mammalian oocytes that can be obtained during a single collection are far fewer (∼1×10^1^ to 1×10^2^) than most other cell types typically cryopreserved such as spermatozoa (1×10^6^ to 1×10^9^) or red blood cells (1×10^7^–1×10^10^)[3]. Mature oocytes are arrested in metaphase of the second meiotic reduction (metaphase II or MII oocytes) and are not capable of normal cell division until fertilization. In this regard, MII oocytes are unlike cell lines which can “survive” cryopreservation by simply compensating for low cryopreservation efficiencies *via* rapid cell division either *in vitro* (*e.g.*, many immortalized cell lines) or *in vivo* (*e.g.*, hematopoietic stem cells). Therefore oocyte cryopreservation has a crucial particularity: each individual cell matters. For most mammalian species studied to date, the overall success of the method (taking into account the cryosurvival rate, the fertilization and the developmental competence of thawed oocytes) is very low; recent publications using human oocytes report developmental competence less than 10% (1%, [4]; 5.6% [5]). Understanding the mechanism of cryodamage would facilitate the development of improved cryopreservation techniques that yield higher survival rates and better developmental competence.

Most cryopreservation techniques currently in use try to prevent cryoinjury (intracellular ice crystal formation, solute effects injury, or both) using equilibrium (*e.g.* slow cooling) or non-equilibrium (*e.g.* vitrification) cryopreservation methods [3], [6], [7]. However, when applied to oocyte cryopreservation, these approaches often lead to decreased cell viability that adversely affects the post-fertilization development of the zygote [8], [9].

The cytoskeletal network in mammalian oocytes has a particular organization of microtubules and microfilaments, as well as intermediate filaments [10], [11], all subject to alteration due to one or more of the steps in cryopreservation. Studies assessing the effects of cryopreservation on the oocyte cytoskeleton have provided limited information. Most have focused on the meiotic spindle [5], [12]–[14] which contains dynamic microtubules [15] that are easily disrupted by cold temperatures and cryoprotectant compounds (reviewed in [16]). Nearly all of those aimed at studying microfilaments have been restricted to qualitative studies on the cortical actin band [17]. More global and quantitative characterization of the post-frozen cytoskeletal network *per se* has not been performed. Since microfilaments control a number of cellular functions [18] and postfertilization events in oocytes [19], in the present work we investigated the relationship between the survival rate and developmental competence of cryopreserved oocytes and those biophysical properties of the cytoplasm that strongly depend on the integrity of the actin cytoskeleton.

In this current series of studies, our first objective was to identify and quantify alterations in the actin cytoskeleton induced by cryopreservation. Cytoplasmic viscosity of human oocytes correlates with their developmental competence [20]. Studies performed on somatic cells show that intracellular viscoelastic properties are strongly affected by cytoskeletal integrity [21]. These observations suggest that intracellular viscoelastic parameters may provide a quantitative measure of cytoskeletal integrity, which, in turn, may reflect the developmental competence of oocytes. We thus studied the intracellular viscoelastic characteristics of fresh and cryopreserved (frozen-thawed) oocytes.

Next, we hypothesized that monomeric G-actin is not affected the same way by cryopreservation stresses (*e.g.* ice crystal formation and exposure to high solute concentrations) as an organized interconnected F-actin network. To test this hypothesis, prior to cryopreservation we disassembled the microfilament network using Latrunculin A (LATA; [22], [23]), a widely employed, highly specific F-actin destabilizing drug, whose molecules form 1∶1 bound pairs with G actin and whose effect is reversible. We evaluated the effect of LATA pretreatment on oocytes by quantitative intracellular viscoelasticity measurements.

Finally, we performed *in vitro* fertilization (IVF) and evaluated the ability to participate in fertilization and the developmental competence of cryopreserved oocytes having been pretreated with LATA, and compared the results with those of control oocytes (i.e. not pretreated with LATA).

## Materials and Methods

### Oocyte collection

Female CD-1 mice (3–4 weeks old) were obtained from Charles River Laboratories (Boston, MA) and housed under a 14 h light/10 h dark cycle in an environmentally controlled room with access to food and water *ad libitum*. Mice were housed for 1–2 weeks prior to initiating superovulation. Pregnant mare serum gonadotropin (National Hormone and Peptide Program, Torrance, CA; 5–6.5 IU) dissolved in sterile saline was injected intraperitoneally, followed by human chorionic gonadotropin (hCG, Calbiochem, La Jolla, CA; 5–6.5 IU) approximately 48 hours later. Thirteen hours post-hCG the mice were euthanized and oviducts were excised and immediately placed into warm Hepes-buffered Tyrode's albumin lactate pyruvate medium (TALP-Hepes) [24] with bovine serum albumin (3 mg/ml; Sigma Aldrich, St. Louis, MO). All solutions of TALP-Hepes were maintained near 37°C during *in vitro* holding. Clutches of cumulus-oocyte complexes were dissected out of the oviducts into TALP-Hepes containing dissolved hyauloronidase (750–1500 IU final concentration; Sigma, St. Louis, MO) and held for 3–4 minutes. Oocytes, freed of cumulus cells, were washed through 3 volumes (∼2 ml each) of TALP-Hepes, and then held in a smaller drop (∼500 µl) of TALP-Hepes covered in sterile mineral oil until further processing. Manipulation of the oocytes was conducted using a fine-pulled glass pipette. We used the minimum number of oocytes in a particular experiment needed to achieve statistical significance. All procedures using animals were approved by the University of Missouri animal care and use committee and conducted in accordance with standards as described in the Guide for the care and use of laboratory animals (National Research Council, Washington DC).

### Bead injection

**Figure 1:**
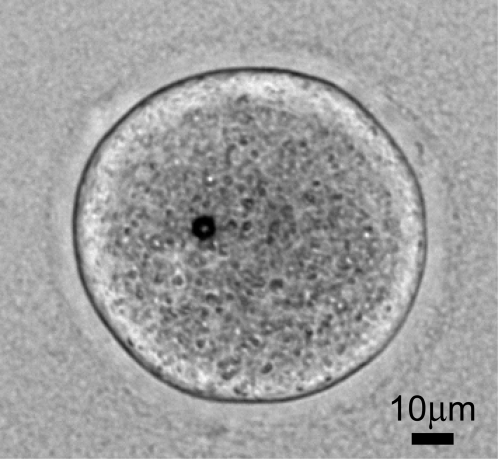
A magnetic bead inside of a mouse oocyte. A photomicrograph of a CD1 mouse oocyte having been injected with a 5 µm superparamagnetic bead, and subsequently embedded in fibrin gel, is shown.

CD-1 mouse oocytes were injected with 5 µm superparamagnetic beads (Dynabeads M-500, Dynal ASA, Oslo, Norway), one bead/cell, from a stock solution containing 2×10^5^ beads/ml in water (Fig. 1). Beads were initially positioned near the center of the oocyte. Approximately 1 µl of the stock solution was transferred to 20 µl of TALP-Hepes, and individual beads were aspirated into a pulled micropipette with an inner diameter of approximately 7 µm. Injection was carried out using piezo-driven (Prime Tech, LTD; Ibaraki, Japan) micromanipulator-supported pipettes (Eppendorf; Hamburg, Germany). After injection, oocytes were returned to TALP-Hepes and held until viscoelastic measurements were carried out. Bead injection was performed within 1 hour after cumulus cell removal for non-frozen oocytes, or within 1 h after dimethylsulfoxide (DMSO) removal for the frozen and thawed oocytes.

### Treatment group assignment for viscoelastic experiments

Injected oocytes from each animal were kept separate from oocytes from other animals and were randomly distributed into four experimental groups as follows: fresh-control group (no treatment); fresh-LATA group [fresh oocytes were incubated in 1 µM LATA (Molecular Probes, Eugene, OR) in TALP-Hepes for ∼45 minutes at ∼37°C]; frozen-thawed group (cells were cryopreserved without LATA); frozen-thawed-LATA group (cells were incubated in LATA as in the fresh-LATA group, subjected to freezing/thawing and finally to thorough washes to eliminate LATA). The LATA solution was prepared in TALP-Hepes by diluting a 100 µM stock solution in DMSO. The frozen-thawed and frozen-thawed-LATA groups were cryopreserved, held in liquid nitrogen for about 24 hours, and then thawed according to the procedures described below.

### Cryopreservation experiments

All chemicals were from the Sigma Aldrich chemical company (St Louis, MO) unless otherwise noted. Oocytes from each female were collected as described above, split into 2 groups, and randomly assigned to the LATA or no LATA treatments. Oocytes were cryopreserved following a published protocol [25] with slight modifications. The solution of the cryoprotective agent (CPA) was prepared by dissolving DMSO (1 M final concentration) and sucrose (0.2 M final concentration) in TALP-Hepes plus fetal bovine serum (10% v/v). Oocytes were incubated in the cryoprotectant solution for ∼30 minutes at 2–4°C [26], [27] by pipetting them into the solution contained within a 250 µl freezing straw being held in an ice bath. The column of medium in the straw was approximately 100 µl in volume. This column was separated from 2 additional columns of medium within the straw with air bubbles. The total volume of medium in the straw was approximately 200 µl. The straws were heat sealed prior to initiating freezing. For the cells assigned to the LATA treatment, the cryoprotectant solution was supplemented with LATA at a final concentration of 1 µM. After the 30 minute CPA equilibration period, the straws were placed into a programmable freezing unit (Planer Kryo 10 Series II; Sunbury-on-Thames, England). The program started at 4°C and cooling proceeded to –6°C at a rate of 2°C per minute; the program then held at this temperature for 10 minutes. Extracellular ice was seeded after the temperature reached –6°C by touching the side of the freezing straw with a cotton–tipped swab having been submerged in liquid nitrogen. After the holding period ended, cooling proceeded to –80°C at a rate of 0.5°C per minute. After reaching –80°C, the straws were plunged into liquid nitrogen and subsequently transferred to a liquid nitrogen dewar for storage. For thawing, the straws were removed from liquid nitrogen and placed into the programmable freezing unit which was initially cooled to –80°C. Warming proceeded at a rate of 8°C per minute from –80°C until 4°C, at which time the straws were removed. The dilution procedure was conducted at room temperature (∼25°C). The solution in the straws was first expelled into a 35 mm Petri dish (Falcon 35–1008; Becton-Dickinson, Franklin Lakes, NJ), and to this solution was added 200 µl of 0.2 M sucrose in TALP-Hepes. After 4 minutes, the oocytes were pipetted out of this solution and transferred to a 50 µl drop of 0.2 M sucrose in TALP-Hepes. After 4 minutes, 50 µl of TALP-Hepes was added to this solution. Four minutes later, 100 µl of TALP-Hepes was added to this solution. Finally, after 4 minutes the oocytes were transferred to 200 µl of TALP-Hepes for a final wash. Injection of the magnetic beads occurred within 1 hour after this final wash. Since the dilutions took place without LATA in the culture medium, this procedure assured that by the time the quantitative experiments were performed, the drug had been washed out and the actin cytoskeleton had sufficient time to recover. We chose to employ the above freeze/thaw protocol primarily for two reasons: 1) most human fertility clinics use slow-cooling methods to cryopreserve human oocytes; 2) the factors utilized in this procedure were developed specifically for mouse oocytes based upon cryobiology theory.

### Magnetic tweezers (MTW) experiments

For viscoelastic measurements, oocytes in each of the four groups (see above) were embedded in a fibrin gel to prevent their movement under the action of the magnetic force, as described below. In a typical experiment, one oocyte was placed on a triangular coverslip in 10 µl of 10 mg/ml bovine fibrinogen solution (in 0.15 M NaCl), covered with mineral oil to prevent evaporation. The fibrinogen solution was then converted to fibrin gel by adding 1–2 µl of bovine thrombin solution (50 NIH units/ml). The gelation time was 1–2 minutes. The triangular coverslip, prepared by cutting 18 mm square No 1½ coverslips (Corning Inc., Corning, NY) was placed in a sample holder, between the two poles of the MTW mounted on the stage of an inverted Olympus IX70 microscope [28]. Several two-second constant force pulses were applied to the cytoskeletal network through the injected magnetic bead with 30 sec pauses between the pulses. This allowed the bead to fully relax between pulses. The cytoskeletal response was monitored through the movement of the bead using video-microscopy. All measurements were performed at locations ranging from 0 (the center of the cell) to 0.85*R*, where *R* is the radius of the cell. Note that this experimental procedure allowed probing the cytoskeleton at various locations and thus average out any variability stemming from the possibly distinct local properties of the cytoskeleton and possible micro-environmental changes caused by the bead. The upper limit of 0.85*R* was chosen to avoid the region in the close proximity of the membrane and the consequent effects due to finite size. The magnetic force, which was constant along the entire bead trajectory within a given experiment, ranged from 22 to 280 pN. The bead trajectory was recorded with a CoolSNAP_fx_ video camera (Photometrics, Tucson, AZ) controlled by Image Pro Express (Media Cybernetics, San Diego, CA), and subsequently tracked with in-house developed particle-tracking software. The hardware (MTW, microscope stage and the video camera) was controlled and integrated using Labview (National Instruments Corp., Austin, TX). Oocytes were kept at 37°C in TALP-Hepes until gel embedding. Viscoelastic measurements were performed at room temperature, and a typical experiment lasted 3–5 minutes (including gelation of fibrinogen). Thus the obtained values may differ from the physiological ones. This however does not affect the conclusions of the present study, as its objective was to compare various treatment groups, all evaluated under identical conditions.

### Modeling and analysis of the viscoelastic data

Each bead trajectory was approximated as a sum of an exponential and a linear function of time: *x*(*t*) = *A*·(1−*e* ^−*B*·*t*^)+*C*·*t*, where *x* is bead displacement, *t* is time and *A*, *B* and *C* are fitting parameters. This model-independent approach provided *τ*, the relaxation time of the cytoplasm (*τ* = 1/*B*), a universal intrinsic property of viscoelastic materials [29].

**Figure 2:**
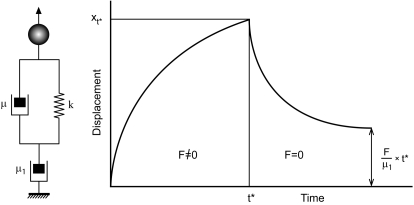
The viscoelastic model for the ooplasmic environment. The ooplasmic environment is modeled as a Voight body (*k*,*µ*) in series with a dashpot (*µ*_1_). The ideal trajectory of the bead under constant force (creep, *F*≠0) and during relaxation (*F* = 0). *F* = magnetic force; *k* = elastic coefficient; *µ* and *µ*_1_ = friction coefficients.

Each bead trajectory was also modeled with the simplest viscoelastic model (Fig. 2) which realistically described the cytoplasmic environment during creep (*F*≠0), as described in ref. [28]. Each oocyte was characterized in terms of elastic and viscous parameters. The elastic (*k*) and friction (*µ* and *µ*_1_) coefficients were determined by fitting the creep part of the bead displacement curve with the solution of the model: 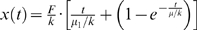, where *F* is the magnetic force. Note that for large *t* (>>*µ*/*k*), *x*(*t*) is determined by the combination of a constant spring-like (i.e. *F*/*k*) and viscous (i.e. *Ft*/*µ*_1_) deformation, the latter characterized exclusively by the cytoskeletal component of cytoplasmic friction, *µ*_1_. For shorter times (i.e. *t*<*µ*/*k*), *x*(*t*) represents a mostly viscous displacement (proportional to 1/*µ*_1_+1/*µ*), quantified in terms of both the cytoskeletal and liquid component of cytoplasmic friction (see Discussion for the physical interpretation of *µ* and *µ*_1_). Comparison of the two expressions for *x*(*t*) leads to *τ* = *µ*/*k*. Similarly, the fitting parameters *A* and *C* can be identified respectively with 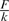 and 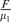. The friction parameters (*µ* and *µ*_1_) can be converted into cytoplasmic viscosity (*η* and *η*_1_) according to the Stoke's formula *η* = *µ*/(6*πR*), where *R* is the radius of the bead.

Since it has recently been noted that the mechanical behavior of living cells shows time-scale invariance [30], [31], we also fit the creep using a power law: *j*(*t*) = *A*(*t*/*t*_0_)*^α^*. Here *j* is the compliance (bead displacement normalized by force), *t*_0_ = 1 s and *A* and *α* are fitting parameters [30], [31].

The quantitative results obtained for the viscoelastic parameters *τ*, *k*, *µ* and *µ*_1_ in the fresh-LATA-treated, frozen-thawed and frozen-thawed-LATA groups were compared with those for the fresh-control group using ANOVA in SAS Statistical Software (SAS Institute Inc., Cary, NC) and the LSD comparison procedure. The data was log-transformed to correct for non-normal distributions.

### *In vitro* fertilization

Sperm capacitation and *in vitro* fertilization (performed as previously described [32]) were conducted in 500 µl drops of human tubal fluid (HTF) medium in organ culture dishes (Falcon 35–3037) covered in sterile mineral oil in a humid incubator at 37°C with a 5% CO_2_/air atmosphere. The medium was made the afternoon before the experiments and equilibrated overnight in the incubator. Approximately 12 h post-hCG injection, sperm were collected from the *vasa defferentia* and the *cauda epididymides* of 10–12 week old males and incubated for approximately 60 minutes prior to transfer to the IVF medium. For control IVF reactions, oocytes were recovered from female mice as described above, the cumulus cells were removed, and the oocytes were placed into media in one of the IVF dishes. For oocytes undergoing IVF after having been frozen with or without LATA, the cells were thawed and washed as described above, then placed in separate IVF dishes. The frozen and thawed oocytes were allowed ∼1 h post-thaw recovery prior to adding sperm. The experimental manipulations were all coordinated such that the sperm (10 µl of the sperm suspension) were added at the same time to the IVF media containing frozen/thawed oocytes and fresh oocytes. After the 5–6 h of sperm/oocyte co-incubation, the presumptive zygotes were removed, washed in TALP-Hepes, and transferred to a 50 µl drop of modified Potassium Simplex Optimized Medium with amino acids (mKSOM^AA^)[33] which had been pre-equilibrated overnight as described for the IVF medium. The following morning, 2-cell embryos were counted and washed through 3 additional drops of mKSOM^AA^ and cultured for 3 additional days (Day 5) prior to counting the number of blastocysts. Thirteen replicates of IVF were performed; the total number of oocytes analyzed for the LATA treatment and no LATA treatment was 256 and 266 respectively.

### Analysis of the IVF data

Data from the IVF experiments were analyzed using the statistical tools in Microsoft Excel. Comparisons between oocyte development with and without LATA treatment were conducted using a paired t-test. For all statistical comparisons, a *p*-value of 0.05 was used as the significance level.

## Results

### Magnetic bead movement through the intracellular environment

**Figure 3:**
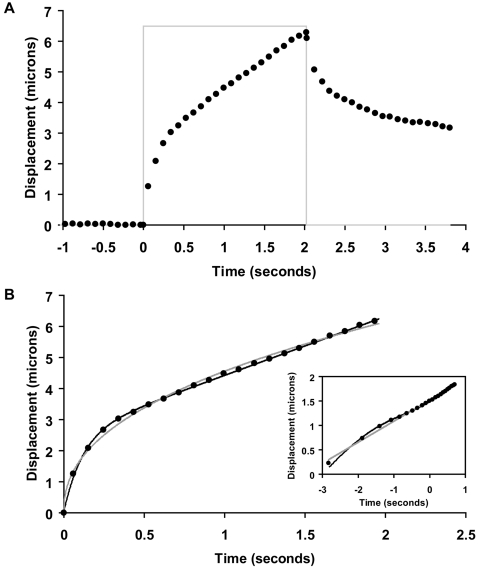
The cytoplasmic trajectory of a magnetic bead. A.Typical bead trajectory under a 2-second constant force pulse (filled circles: bead position; gray line: magnetic pulse; “0” on the time axis represents the moment when the force pulse was applied). B. Best fit to the trajectory during creep (*F*≠0). Filled circles: experimental data points; black line: best fit with the linear viscoelastic model; gray line: best fit with the power law. Inset: bead trajectory on a log-log scale. The numbers along both axes are powers of e, the base of natural logarithm.

To analyze the viscoelastic properties of fresh and cryopreserved oocytes, we injected magnetic beads into the ooplasm (Fig. 1) and followed their movement under a constant force exerted by a magnetic tweezers. The recorded trajectories were fit using models of linear viscoelasticity [34] and a power law function [30], [35] (see Materials and Methods). Results in Fig. 3 show that both approaches provide an excellent description of bead movement. The power law form reflects the known characteristics of viscoelastic materials: they possess a spectrum of relaxation times. The fact that the adapted model of linear viscoelasticity provided similarly good results suggests that this spectrum is dominated by one (i.e. largest) *τ* value. Since the description of viscoelasticity in terms of viscosities and elastic constants is conceptually simpler, in what follows we employ the linear model (although for completeness we also provide numerical values in Table 1 for the power law fit).

**Table 1:** Viscoelastic coefficients.

	Fresh-control untreated	Fresh-LATA	Frozen-thawed	Frozen-thawed- LATA
*τ* (sec; geometric mean±geometric SE)	0.153±0.001	0.129±0.002*	0.204±0.004*	0.162±0.003
*k* (pN×µm; mean±SEM)	61.6±9.4	52.7±10.4	25.7±2.5*	68.6±8.7
*µ* (pN×s/µm, mean±SEM)	9.2±1.2	6.3±0.9*	5.3±0.5*	11.4±1.7
*µ*_1_ (pN×s/µm, mean±SEM)	152.4±21.5	98.3±16.9*	46.2±5.2†	144.5±22.9
*α* (mean±SEM)	0.383±0.009	0.399±0.017	0.471±0.02†	0.417±0.019
*A* (µm/pN, geometric mean±geometric SE)	0.030±0.001	0.041±0.001	0.056±0.003†	0.027±0.001

* *p*<0.05 compared to the fresh-control group.

† *p*<0.01 compared to the fresh-control group.

### LATA treatment and cryopreservation alter physiological intracellular viscoelastic properties

**Figure 4:**
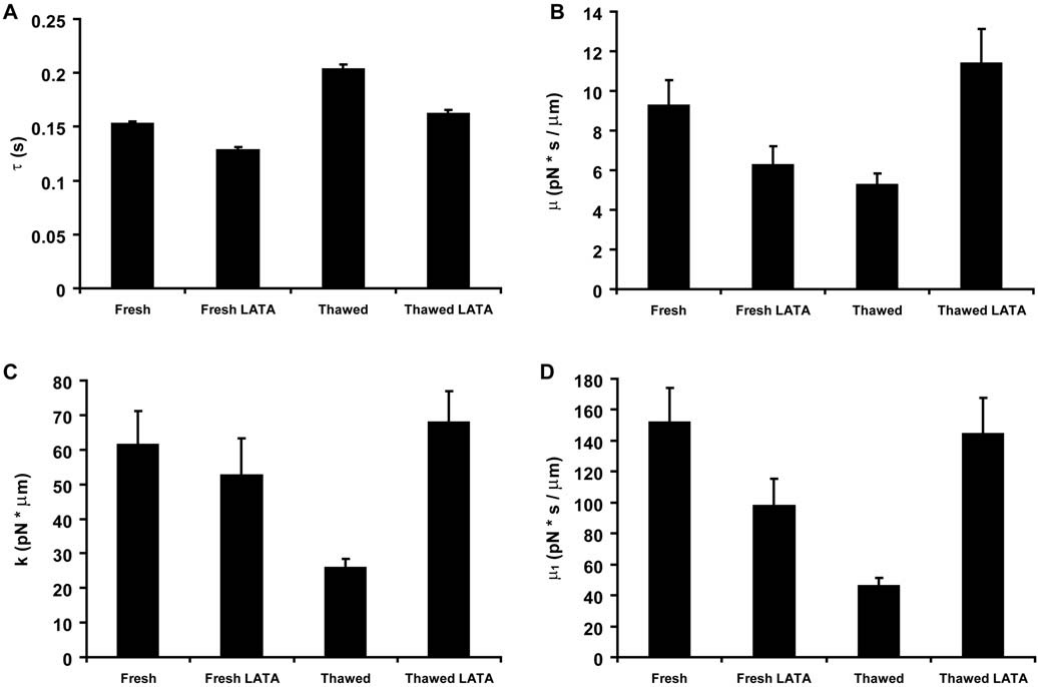
The oocyte viscoelastic parameters. Viscoelastic parameters before freezing without (Fresh, n  =  29 cells) or with LATA treatment (Fresh-LATA, n  =  28 cells) and after freezing without (Thawed, n  =  16 cells) or with (Thawed LATA, n  =  19 cells) LATA pretreatment. (A) Relaxation time. (B) Friction coefficient *µ*_1_. (C) Elastic coefficient *k*. (D) Friction coefficient *µ*. All viscoelastic parameters show significant statistical difference (see *p* values in Table 1) between the Fresh and Thawed groups and most show statistical difference between Fresh and Fresh LATA groups. There is no significant statistical difference between the Fresh and the Thawed LATA groups. Bars represent geometric mean±geometric SE (panel A) and mean±SEM (panels B, C and D).

As the data in Table 1 show, LATA treatment or freezing of oocytes lead to marked changes in material properties, in particular, the relaxation time *τ* (Fig. 4A). As expected, LATA renders the cytoskeleton less rigid, more fluid and responsive (thus smaller *τ*). On the other hand freezing results in a more rigid, less responsive and adaptive cytoskeleton (thus larger *τ*). Note that both treatments lead to the decrease of all the other viscoelastic parameters (*µ*, *µ*_1_, *k*), with the decrease being smaller in the fresh-LATA-treated than in the frozen-control.

### LATA treatment prior to cryopreservation preserves physiological intracellular viscoelastic properties

The relaxation time in the frozen-thawed LATA pre-treated group was not statistically different from the one in the fresh-control group (Table 1 and Fig. 4A). Similarly, *µ*, *µ*_1_ and *k* (as well as the power law parameters) were not significantly different between the fresh-control and frozen-thawed-LATA groups (Table 1 and Fig 4B–D). Note that in light of our experimental procedure (see Materials and Methods), the small error bars in Fig. 4 suggest that even if the magnetic bead caused modifications in its microenvironment, these were of similar magnitude at distinct locations within the cell, and thus likely not to affect the conclusions of our comparative studies.

### LATA treatment prior to cryopreservation improves the survival rate and developmental competence of frozen-thawed oocytes

**Figure 5:**
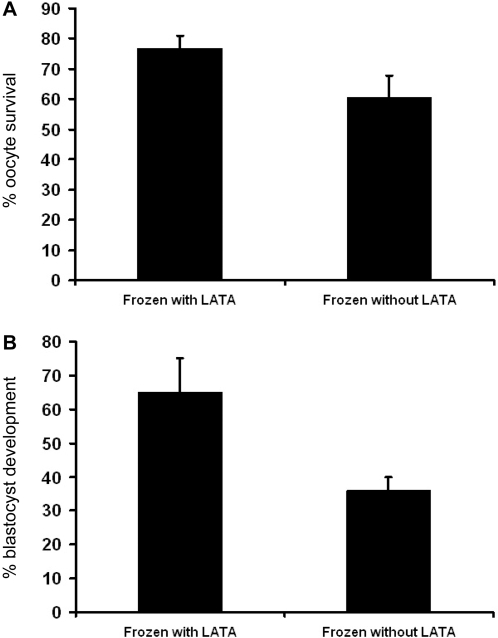
Oocyte survival after cryopreservation. (A) The percentage of mouse oocytes that survived slow-cooling with (left) and without (right) LATA pretreatment. LATA pretreatment increased the cryosurvival rate by 26.2% (*p*<0.05). (B) The percentage of blastocysts developed from 2-cell embryos with (left) and without (right) LATA pretreatment. LATA pretreatment increased developmental competence by 81% (*p*<0.05). Error bars represent the SEM.

LATA pre-treatment resulted in a 26.2% increase in the number of oocytes that survived cryopreservation when compared with the non-treated group (Fig. 5A): 77±4% versus 61±7% respectively (mean±SEM, *p*<0.05). Survival was evaluated by comparing oocyte morphology upon freeze-thaw with that of fresh cells (based on microscopic observation).

Of those oocytes that survived cryopreservation, cleavage to the 2-cell stage did not differ between frozen-control and frozen-thawed-LATA groups: mean  =  29% for each group. (The low fertilization rates of cryopreserved oocytes, compared to 80% in fresh oocytes, were likely due to zona hardening in the strain used, as previously described [36], [37]). Among those oocytes undergoing initial cleavage, the rate of blastocyst development was significantly higher for oocytes exposed to LATA prior to freezing: 65±10% versus 36±14% (mean±SEM, *p*<0.05; Fig. 5B). To determine the possible effect of LATA on IVF in non-cryopreserved oocytes, we treated fresh oocytes with LATA and subsequently eliminated it prior to IVF. When compared with the untreated group, no differences in fertilization and subsequent development to the blastocyst stage were observed (p>0.05; data not shown).

## Discussion

The aim of this study was to investigate the mechanisms associated with cryodamage and apply this new knowledge to improve on the survival rate and developmental competence of cryopreserved oocytes. Our approach was based on (1) the observation that cryopreservation diminishes actin cytoskeleton integrity in apparently normal looking oocytes (upon optical inspection) and (2) the hypothesis that cryopreservation, in the absence of a microfilament network, causes less damage to the oocyte than in its presence. Thus, we reversibly dismantled the F actin network of mouse oocytes prior to their freezing. The efficacy of this method was assessed by quantitative measurements of intracellular viscoelastic properties and IVF. Viscoelasticity measurements were carried out using a magnetic tweezers: magnetic beads injected into oocytes (Fig. 1) were displaced under controlled force and their trajectory was recorded (Fig. 3). Viscoelastic parameters were determined by modeling these trajectories with standard models of viscoelasticity (Fig. 2 and 3). IVF was performed on both control and LATA treated oocytes and the outcomes compared at different developmental stages (Fig. 5).

The characterization of the physical properties of the cytoplasmic environment through the displacement of magnetic beads is a powerful method with a long history [38]–[44]. The reported numerical values of the viscoelastic parameters show a strong dependence on cell type and the specific method used. Our results for fresh mouse oocytes are much closer to those found in sea urchin eggs [42] than to those in various somatic cells [38], [41], [43], [44].

The results from our quantitative study were interpreted in terms of three characteristic cytoplasmic quantities (Fig. 2): the cytoskeletal (*µ*_1_) and liquid (*µ*) friction constants and an elastic coefficient (*k*). As the bead starts moving, it commences to locally deform the cytoskeleton. This elastic deformation continues until *t*≈*τ*, where the time scale *τ* = *µ*/*k* (another characteristic cytoplasmic quantity) is determined by both the viscous and elastic properties of the environment. During this time, bead displacement (proportional to1/*µ*+1/*µ*_1_) is dominated by the liquid component of cytoplasmic viscosity (1/*µ*+1/*µ*_1_≈1/*µ*; Table 1). The origin of *µ* is mostly in the viscous liquid properties of the cytoplasm (for fresh oocytes, the associated viscosity *η*≈10^−1^ Pa×s, is ∼100 times larger than that of water), whereas the origin of *k* is in the elasticity of the cytoskeletal filaments. Once the cytoskeletal mesh is locally maximally stretched (i.e. *t*>*µ*/*k*), the bead's total displacement is dominated by the combination of this stretch (i.e. *F*/*k*) and the deformation due to the viscous properties of the oocyte cytoskeleton, whose origin is in the breakage of cross-links between the bead and the mesh [40]. Previously, it has been pointed out that the in vitro removal of cumulus cells from ovulated oocytes using hyaluronidase (as was done in this study and most oocyte cryopreservation methods), alters the cell-cell and cell-zona interactions, increases the porosity of the zona pellucida and enhances the release of perivitelline factors. All these effects could change the cytoplasmic properties of the oocyte [45]. Hence, we cannot be certain that the parameters listed in Table 1 are identical to those of ovulated oocytes not having been enzymatically treated. Their variation across the treatment groups, as shown in Table 1, is however informative of the consequences of oocyte cryopreservation under the conditions investigated in this study, which are relevant in practical applications.

Cryopreservation in the absence of LATA treatment (frozen-thawed group) resulted in considerable increase of the relaxation time and reduction of all three viscoelastic parameters (Table 1 and Fig. 4). This finding is consistent with the interpretation of these quantities given above. If the freeze-thaw process indeed leads to the fragmentation of the cytoskeleton and thus to loss in the extent of its interconnectedness, then both its viscous and elastic characteristics are expected to be diminished. LATA pre-treatment reversed this trend: the three viscoelastic constants and the relaxation time do not significantly differ from their corresponding values for fresh oocytes (Table 1 and Fig. 4). Even though both cryopreservation and LATA treatment alone induce the disruption of the cytoskeletal network, the two mechanisms are quite different. First, in the former case *a priori* all components of the cytoskeleton are affected, whereas in the second case only the F actin assembly is altered. Second, changes in cytoplasmic viscoelastic properties are mostly irreversible upon cryopreservation, whereas they are mostly reversible following LATA treatment [41]. Results listed in Table 1 explicitly show the quantitative effect of the two treatments on the viscoelastic parameters of oocytes (compare the fresh-LATA and frozen-thawed groups). Whereas both treatments reduce the values of *µ*, *µ*_1_ and *k*, the effect of cryopreservation is more dramatic than that of 1 µM LATA treatment. Furthermore, not all parameters are equally affected: *k* is diminished much more by cryopreservation than LATA, while the change in *µ* is similar. As a consequence, *τ* is respectively increased and decreased by the two treatments, a result to be expected on general grounds; cryopreservation and LATA render the cell respectively less and more responsive to perturbation caused by the moving magnetic bead. (Note that the changes in the power law parameters, upon the destabilization of the actin network of the fresh cells, as listed in Table 1, are consistent with earlier results obtained on somatic cells [31].)

The above results support the hypothesis that the effect of cryopreservation on the F actin mesh is much more critical than on the collection of G actin molecules. Our findings imply that freezing makes irreversible changes in the association of G actin molecules, which prevents the microfilament network from re-establishing its physiological viscoelastic properties upon thawing. The disassembly of the network prior to cryopreservation and the removal of LATA after thawing, on the other hand, allow the formation of a *de novo* microfilament network with no “memory” of cryopreservation. Furthermore, these findings also suggest that, at least in the mouse model, the effect of freezing is more dramatic on the actin mesh than on the other components of the cytoskeleton.

Another possible interpretation of the effect of LATA on oocyte survival is directly related to cell membrane repair. Indeed, immediately upon thawing, a higher proportion of oocytes frozen in the presence of LATA than in its absence had an intact oolemma, as observed by light microscopy. Plasma membrane disruption has long been known to be one of the principle mechanisms of cell cryodamage [46]. Our results are consistent with previous findings demonstrating an enhanced ability of cells to repair cell membrane wounds upon the disassembly of their actin cytoskeleton [47]–[49].

Microfilaments play a significant role in oocyte maturation and fertilization events (reviewed in ref. [50]). Qualitative studies focusing on the cortical microfilaments have shown that factors associated with cryopreservation (*e.g.* exposure to the cryoprotectant) can cause disruption of the microfilament network in mammalian oocytes (reviewed in ref. [16]), the magnitude of the effect depending on the particular CPA used, or species from which the oocyte is derived [17], [51]–[53]. One particular organelle strongly associated with the microfilament network (through the cortical actin mesh) is the plasma membrane. If these two structures are differentially affected by freezing, the former (due to its decreased elasticity) may impose increased stress on the latter and possibly cause permanent damage in its structure. This is supported by the findings of Eroglu et al. [54] who examined the effect of cryopreservation on the cytoskeleton and early post-fertilization events in mouse oocytes. When analyzed immediately after thawing the cortical actin band displayed dramatic alterations. However, the authors also observed that these effects were much reduced following incubation after thawing for 1 hour at 37°C. Furthermore, they reported that IVF, immediately after removal of CPA, resulted in a significant decrease in the fertilization rate and aberrant dynamics of cytoskeleton-dependent events, whereas oocytes inseminated after the post-thaw incubation displayed improved fertilization rates (assessed by oocyte activation following sperm entry) and cytoskeletal architecture (assessed by immunostaining). In another study, Saunders and Parks [17] found that neither the cortical actin band nor cytoplasmic microfilaments of bovine oocytes could recover from the damage caused by cryopreservation, despite extensive post-thaw incubation. Consistent with this observation, after IVF, the number of frozen-thawed oocytes undergoing cleavage was dramatically reduced relative to fresh oocytes. Dobrinsky et al. [55] determined that exposure of porcine early hatched blastocysts to cytochalasin B prior to freezing resulted in improved post-thaw viability, suggesting that the F actin network is a target of cryoinjury across different cell types.

These and similar findings motivated us to reversibly dismantle the microfilament network prior to cryopreservation. We surmised that exposure to LATA could prevent stress from being transmitted to and from the membrane through the actin network. As a result, the membrane may become more flexible and better tolerate the osmotic imbalance arising in the course of freezing and thawing. Conversely, intracellular structures would be exposed less to stress caused by membrane tension variations. (In our study, all cryopreserved oocytes underwent a 1 hour post-thaw recovery prior to insemination.)

To the best of our knowledge, ours is the first quantitative study to assess the effects of cryopreservation on microfilaments in mature oocytes. We have shown that freezing/thawing results in a state characterized by a significant alteration of cytoplasmic viscoelastic properties, which do not appear to be fully reversible by post-thaw incubation in culture. While we could accurately characterize this state, our method does not allow determining with certainty whether it is freezing or thawing that is primarily responsible for the observed changes. Our findings indicate that reversible depolymerization of the actin network prior to cryopreservation largely preserves physiological viscoelasticity. It is known that F actin interacts with the other major cytoskeletal components [56] (i.e. intermediate filaments [57], microtubules [58]) and therefore could be affected by LATA treatment. Thus our results are not necessarily specific to actin, rather reflect the importance of overall cytoskeletal integrity. Our observations then suggest that the reversible disruption of the cytoskeleton (through any of its interacting components) prior to cryopreservation, may confer a protective effect and lead to considerable improvement in the survival rate and developmental competence of frozen-thawed mouse oocytes. In the present work we used LATA treatment, and developmental competence was assessed by the relative number of oocytes reaching the blastocyst stage following IVF. Because of some differences in oocytes across taxa [59], the results from this study may not be directly applicable to oocytes from other mammals, but do suggest that further investigations are warranted. These results demonstrate that protecting the oocyte cytoskeleton from damage during cryopreservation is important to the development of the early embryo, particularly beyond the first cleavage division. Further development to term may also be differentially affected by treating oocytes with LATA before cryopreservation, and is another important factor to evaluate, particularly before such a treatment is applied to human oocytes. Nevertheless, our results point to a new role for LATA as a cryoprotective agent, in particular in case of oocytes.
